# Detecting Conversation Topics in Recruitment Calls of African American Participants to the All of Us Research Program Using Machine Learning: Model Development and Validation Study

**DOI:** 10.2196/65320

**Published:** 2025-07-17

**Authors:** Priscilla Pemu, Michael Prude, Atuarra McCaslin, Elizabeth Ojemakinde, Christopher Awad, Kelechi Igwe, Anny Rodriguez, Jasmine Foriest, Muhammed Idris

**Affiliations:** 1 Department of Medicine Morehouse School of Medicine Atlanta, GA United States; 2 Clinical Research Center Morehouse School of Medicine Atlanta, GA United States; 3 Wexner Medical Center The Ohio State University Colombus, OH United States; 4 Department of Public Health Education Morehouse School of Medicine Atlanta, GA United States; 5 Human-Centered Computing School of Interactive Computing Georgia Institute of Technology Atlanta, GA United States

**Keywords:** recruitment, clinical trials, diversity, trust, precision medicine

## Abstract

**Background:**

Advancements in science and technology can exacerbate health disparities, particularly when there is a lack of diversity in clinical research, which limits the benefits of innovations for underrepresented communities. Programs like the All of Us Research Program (AoURP) are actively working to address this issue by ensuring that underrepresented populations are represented in biomedical research, promoting equitable participation, and advancing health outcomes for all. African American communities have been particularly underrepresented in clinical research, often due to historical instances of research misconduct, such as the Tuskegee Syphilis Study, which have deeply impacted trust and willingness to participate in research studies. With the US population becoming increasingly diverse, it is crucial that clinical research studies reflect this diversity to improve health outcomes. However, limited data and small sample sizes in qualitative studies on the inclusion of underrepresented groups hinder progress in this area.

**Objective:**

The goal of this paper is to analyze recruitment conversations between research assistants (RAs) and potential participants in the AoURP to identify key topics that influence enrollment. By examining these interactions, we aim to provide insights that can improve engagement strategies and recruitment practices for underrepresented groups in biomedical research.

**Methods:**

Our study design was an observational, retrospective approach using machine learning for content analysis. Specifically, we used structural topic modeling to identify and compare latent topics of conversation in recruitment calls by Morehouse School of Medicine RAs between February 2021 and April 2022 by estimating expected topic proportions in the corpus as a function of enrollment and participation in AoURP.

**Results:**

In total, our model estimated 45 topics of which 12 coherent topics were identified. Notable topics, that were more likely to occur in conversations between RAs and participants that enrolled and participated, include closing or following up to schedule an appointment, COVID-19 protocols for in-person visits, explaining precision medicine and the need for representation, and working through objections, including concerns about costs, insurance, care changes, and health fears. Topics among potential participants who did not enroll include technical challenges and describing physical measurement visits (eg, collection of basic physical data, such as height, weight, and blood pressure).

**Conclusions:**

Using an approach that leverages machine learning to identify topical structure and themes with limited human subjectivity is a promising strategy to identify gaps in, and opportunities to improve, the recruitment of underserved communities into clinical trials.

## Introduction

### Background and Significance

Advancements in science and technology sometimes worsen the gaps in health outcomes [1-3]. Lack of diversity in clinical research contributes to this by negatively influencing the diffusion of such innovations in communities that are not well represented. The projected demographic shift in the US population underlines the urgency for clinical research studies to reflect the diversity of the affected population [4].

The All of Us Research Program (AoURP) is a national initiative aimed at advancing precision medicine by building a diverse and inclusive research database [5]. A core focus of the program is increasing engagement, enrollment, and retention of underrepresented in biomedical research (UBR) populations, including African American, Hispanic or Latino, and rural communities. African American communities, for example, have faced a long history of exclusion from clinical and biomedical research, with systemic barriers such as limited access to health care, underrepresentation in recruitment efforts, and mistrust stemming from unethical practices like the Tuskegee Syphilis Study [6]. The program uses targeted outreach strategies, such as community partnerships, culturally tailored communication, and participant-centered approaches, to address historical barriers to research participation and foster trust. These efforts are designed to ensure the cohort reflects the diversity of the US population, enabling research that benefits all communities equitably.

A 2022 report from the National Academies of Medicine and Science, “Building Research Equity for Women and Underrepresented Groups” [7] recommended a holistic approach with multiple levels of intervention to reach the goal of inclusiveness in clinical research. One significant level is the role of interaction between the research team and the community with a focus on engagement [7]. These interactions are pivotal, as they directly influence potential participants’ perceptions of the study, trust in the research process, and willingness to participate [7]. The 2022 National Academies of Medicine and Science report highlighted the dearth of critical data about facilitators of successful inclusion of underrepresented groups in clinical trials [7-9]. Research shows that information exchange between members of the clinical research team and potential study participants or volunteers critically influences the research volunteer’s journey from awareness to enrollment [7,10,11]. Current recruitment strategies rely on a combination of outbound and inbound phone calls, community events, and digital outreach, but these interactions are typically not recorded, making analysis of recruitment effectiveness reliant on retrospective assessments rather than direct examination of conversation topics in real-time. Moreover, conclusions drawn from qualitative analyses of small sample sizes and limited examination of offers for clinical trial participation beyond financial and nonfinancial incentives hinder the field [12,13].

### Objective

While many studies have identified barriers to clinical trial enrollment, little is known about what actually happens at the point of recruitment. The goal of this analysis is to contribute to this body of research by leveraging automated content analysis methods to examine conversations during recruitment calls with limited human subjectivity. Specifically, we use structural topic modeling (STM) to explore differences in conversations between research assistants (RAs) and community members from the greater Atlanta metro area recruited for participation in the AoURP during the COVID-19 pandemic.

## Methods

### Study Design

Potential participants in the AoURP contribute their biological, health, behavioral, and environmental data. The data collected in the AoURP includes participant-provided information (PPI) from surveys, available electronic health records (EHRs) with participant permission, baseline physical measurements (eg, height and weight), and biospecimens (eg, blood, urine, and saliva). DNA is isolated from the biospecimen samples for genetic testing. Additionally, the program may also collect data from passive mobile and digital health devices, sensors, and mobile apps.

### Selection of Participants

#### Inclusion Criteria

Eligible AoURP participants must be 18 years or older, currently living in the United States or US territory, and have the legal authority and ability to give informed consent.

#### Exclusion Criteria

Adults who do not have the ability to give informed consent, children younger than 18 years of age, and individuals incarcerated at the time of enrollment are ineligible to participate in AoURP.

### All of Us Southeastern Enrollment Center

Individuals were enrolled through participating health provider organizations or designated venues. The Southeast Enrollment Center (SEEC) is a health provider organization partner of the AoURP consisting of the University of Miami Miller School of Medicine, University of Florida College of Medicine, Emory University, and Morehouse School of Medicine (MSM). Strategically located in a region of the United States with high racial and ethnic diversity, the center is enrolling over 100,000 diverse participants, or approximately 10% of the >1 million individuals targeted by the AoURP nationwide. As of December 2022, the SEEC has successfully enrolled a cohort with 76.23% identifying as UBR populations, including 345 (out of 449) participants who identified as Black or African American [14]. This is a significant contribution given that African Americans have historically been underrepresented in clinical research; for example, in oncology trials, African American men and women make less than 5% and 3% of participants, respectively [15].

### The Setting of Recruitment and Procedures

Recruitment efforts used multiple strategies to reach potential participants. The primary method of recruitment was phone calls, supplemented by web-based or mobile outreach, and where feasible, in-person site support. These approaches help maximize participant engagement and accessibility. Before enrollment, research staff screen and contact participants to ensure they are capable of providing informed consent. During this process, individuals receive clear information about the study and are explicitly informed that their decision to participate does not impact their health care or medical treatment.

All participants complete a standardized electronic informed consent process to ensure uniformity and adherence to ethical guidelines. To accommodate diverse participants, the informed consent process is self-paced, allowing individuals to review materials at their convenience through web-based or mobile platforms. Consent materials are available in English and Spanish, with institutional review board–approved translations to ensure accuracy. For individuals who require additional support, trained staff are available to assist either in person at designated sites or remotely via the All of Us Support Center.

### RA Recruitment

A total of 13 research staff were enlisted and trained to support recruitment efforts during the study period of February 2021 to April 2022. This included seven RAs in their 20s, three RAs in their 30s, and three RAs in their 50s. A total of 12 (92%) RAs were female and one male. Most RAs (12 out of 13) were Black or African American and one Hispanic. One RA completed an associate degree, five RAs a bachelor’s, three RAs an MPH, and three RAs MDs.

### Recruitment Calls

While initially conducted using a combination of face-to-face and via phone calls, the COVID-19 pandemic required us to digitize our recruitment efforts fully. Phone calls were facilitated using a cloud contact center solution called 3CLogic. Transcription was enabled by integration with a commercial speech analytics platform, ObserveAI. Contacts and associated phone numbers in 3Clogic are sourced from a proprietary platform called Engage. RAs are trained separately on Engage and 3Clogic. Call result outcomes were logged as Not Available (if the person you are calling is not available, no one answers the phone, no voicemail, or voicemail full), Wrong Number (if you can verify the number is incorrect), Voicemail (if you left a voicemail message), Scheduled (you have scheduled the person for a visit), Not Interested (potential participant does not want to participate in the AoURP), Do Not Call (potential participant does not want to receive any more calls about the AoURP), and Follow Up (potential participant requests a call at a later date).

### Data Analysis

#### Levels of Participation

There are 4 levels of participation in the AoURP. Interested individuals are potential participants who have provided contact information to receive updates about the program. Registered individuals are participants who have created an account on the AoURP platform but have not yet completed the informed consent process. A registered individual has a unique AoURP participant identifier. Consented individuals have met the eligibility and inclusion criteria and have completed the primary informed consent process. They may also have completed the optional Health Insurance Portability and Accountability Act authorization or EHR consent and genomic return of results (gRoR) consent. Participants are individuals who have completed the primary informed consent process and are eligible to complete the Health Insurance Portability and Accountability Act authorization or EHR consent, gRoR consent, surveys specific to Basics, Lifestyle, and Overall Health PPI modules, and provide physical measurements and biospecimens. Core participants have completed all core components (ie, registration, EHR or gRoR consent, and PPI modules) and provided physical measurements and biospecimens. They may also contribute other data, such as digital health data from sensors and wearables, if available. For the purposes of analysis, any participant who has created an account, completed informed consent, and completed at least one survey or in-person visit, is considered to have “participated at any level.”

#### STM

Following an approach outlined in Idris et al [16], we used STM to analyze the content of potential research volunteer and RA conversations. STM is an advanced machine learning technique designed to uncover themes within a collection of documents or transcripts. It operates on the principle that certain words are linked to specific topics, as demonstrated in previous studies [17,18]. Formally, each utterance in a conversation is represented as a vocabulary multiset with corresponding word counts, also known as a document-term matrix. In this matrix, each word has a probability associated with belonging to a particular topic. By viewing a conversation as a random mixture of topics—each defined by a set of characteristics, high-probability words—we can infer latent topics that may not be immediately apparent.

Using STM allows us to incorporate document-level metadata, such as the context of the conversation, the participants involved, and the timing of interactions. This enriches the model, making it more nuanced and capable of capturing the complexity of real-world conversations. Furthermore, STM’s ability to handle large and diverse datasets makes it particularly suitable for analyzing the varied and dynamic nature of volunteer-RA dialogues.

Our goal in using STM is to uncover underlying patterns and themes that provide valuable insights into the nature of these interactions. Understanding these latent topics can enhance communication strategies, improve volunteer engagement, and identify areas for training and development for RAs. Overall, this approach not only improves the interpretability and relevance of the generated topics but also aligns the findings with the underlying structure of the data, thereby facilitating more informed and actionable conclusions. We used STM in our analysis for three main reasons. First, STM allows the incorporation of document metadata such as participant characteristics into the analysis of latent topics through a generative process that models the relationship between metadata and the proportion of a specific topic in each utterance [19]. This enables us to plot metadata or topic relationships by estimating parameters, including the expected proportion of an utterance belonging to a topic as a function of a covariate, or a first difference estimate, where topic prevalence for a particular topic is contrasted between 2 groups (eg, enrolled or participated vs not enrolled). Second, by incorporating additional contextual information, STM enhances the interpretability and relevance of the generated topics. This results in more coherent and meaningful topics that align with the underlying data structure, thereby facilitating the drawing of insightful conclusions. Finally, using built-in functionality in the STM R package, we avoided subjective decisions on the number of topics by using a data-driven approach [20]. This approach helped eliminate human-based bias in our analysis by identifying words that appear in a document only if the document pertains to a specific topic.

### Ethical Considerations

Participants provided informed consent during the primary data collection process, which included authorization for the use of their data or samples in future research studies, as approved by the institutional review board (protocol #2016-05). All data used in this study were deidentified to ensure the confidentiality and privacy of participants. Participants in the AoURP receive compensation as an acknowledgment of their time and effort. Participants received US $25-$50 for completing initial enrollment procedures and additional compensation for follow-up activities, including such as biospecimen collection, physical measurements, or surveys.

## Results

### Overview

**Figure 1:**
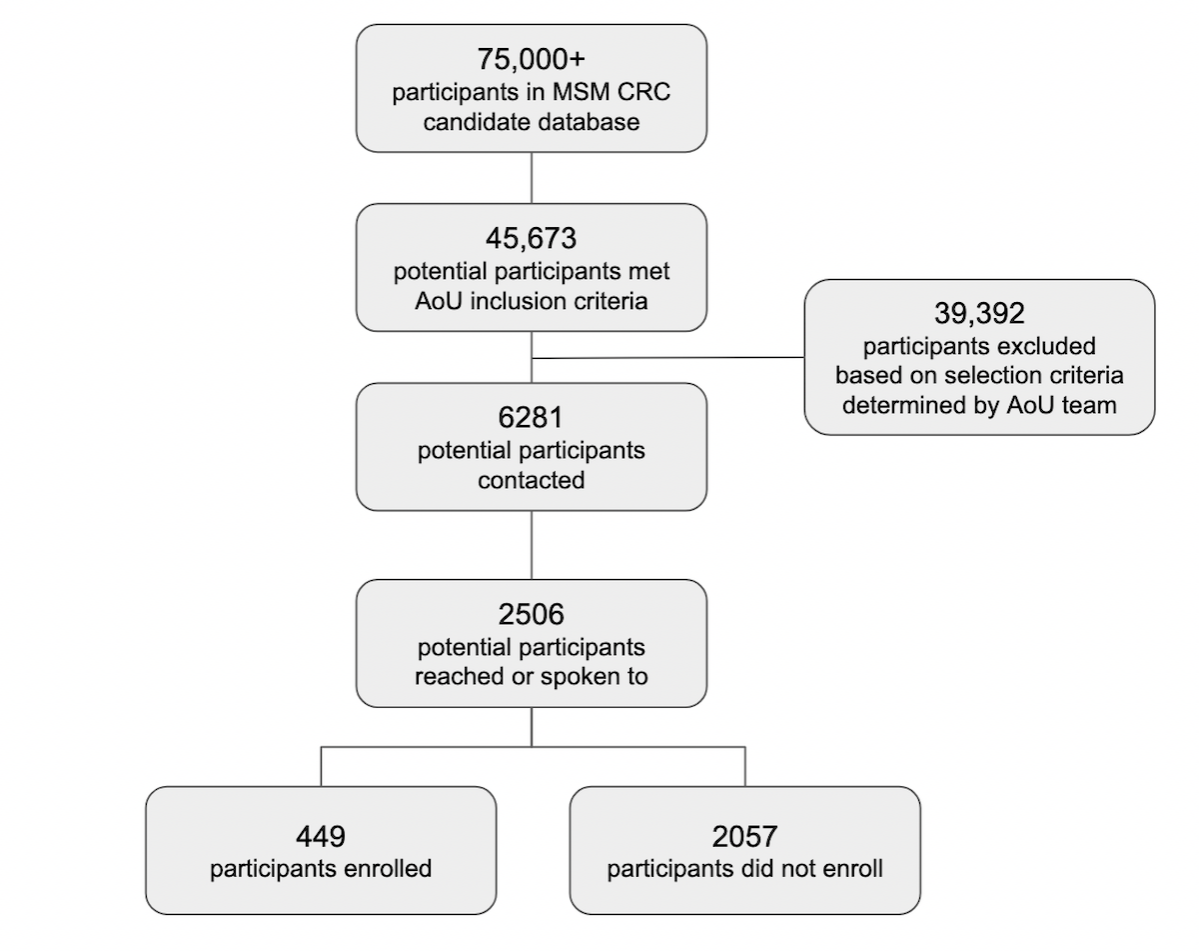
Flowchart of research participants contacted, reached, and enrolled. AoU: All of Us; CRC: Clinical Research Center; MSM: Morehouse School of Medicine.

Table 1 provides the demographics of participants recruited to the AoURP by MSM and compared to other institutions in the Southeastern enrollment center. Figure 1 provides a flowchart of how many participants were contacted, reached, and enrolled between February 2021 and April 2022. In total, RAs made 31,741 calls comprising 4350 conversations with an average of 33.92 (SD 54.66) completed talk turns (eg, a single spoken statement by one speaker before the other speaker responds). The average number of calls per participant was 2.90 for the enrolled group and 1.48 for the not enrolled group. The average length of conversation was 64.28 talk turns for the enrolled group and 25.23 for the not enrolled group, suggesting that on average, longer conversations between RAs and potential participants are associated with a higher likelihood of enrollment.

**Table 1:** Underrepresented in biomedical research of core participants recruited by All of Us southeastern enrollment center (2018-2022; N=29,402).

	Morehouse School of Medicine, %	Southeastern Enrollment Center, %
Overall	97.26	76.23
Ancestry	92.54	66.47
Age	14.02	23.05
Gender	0.03	0.04
Sexual and gender minority	13.54	10.10
Income	53.24	25.31
Education	18.31	9.60
Geography	0.54	3.52

### Structural Topic Model

**Figure 2:**
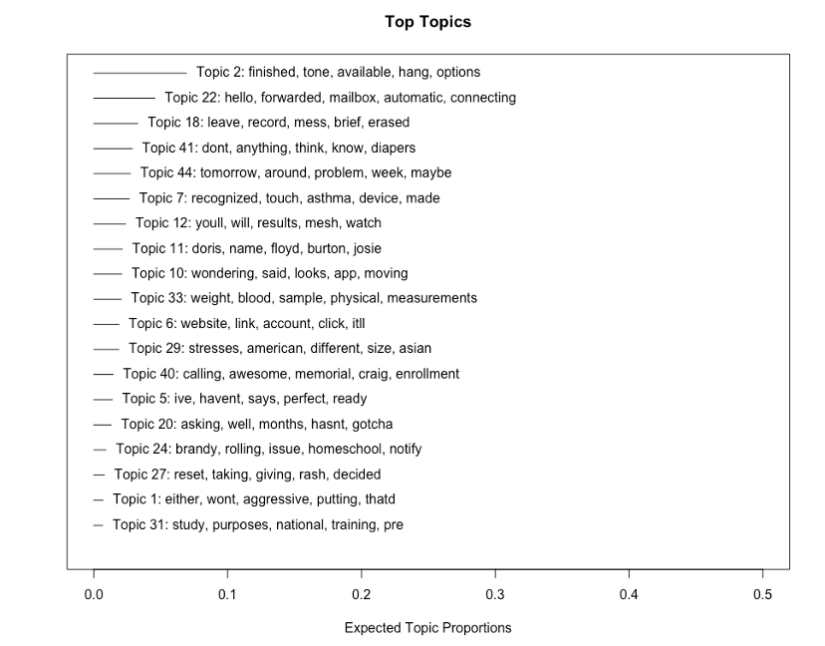
Initial topics ranked by expected proportions with the top 5 associated words.

In total, our corpus included 106,077 talk turns, 4911 terms (eg, a unique word), and 383,763 tokens (eg, every occurrence of a word, including repetitions across different sentences and documents). A total of 45 topics were estimated. In line with common practice, our approach to assigning labels to topics began by reviewing topic model outputs to identify potential topics based on the coherence of characteristic word forms [21]. We then reviewed the high-scoring conversations for each topic to determine topic labels. High-scoring conversations refer to talk turns with the strongest associations to a given topic, as determined by the topic model’s relevance and coherence scores. Where there was a clear topical theme in the first N talk turns, we validated the topic label. In general, a clear theme emerged within the first 25 talk turns. A review of high-scoring talk turns was completed collaboratively by 2 reviewers (PP and MI) until a consensus on topic labels was reached. Of the 45 topics estimated, 12 coherent topics emerged as presented in Textbox 1. Figure 2 plots these initial topics ordered by their expected topic proportions, that is, the expected proportion of the corpus that belongs to each topic, along with the top 5 words associated with each topic. Topics 2 and 22, which clearly reflect automated voicemail prompts have the highest prevalence in the transcripts, which is as expected given the practice of cold calling where most calls go unanswered.

**Coherent topic identified from the Corpus of All of Us recruitment transcripts with top five associated words.**

**Topic 2**

- Automated message prompt

**Topic 5**

- Following up to complete participant surveys

**Topic 6**

- Instructions or struggles accessing the participant portal

**Topic 7**

- Scripted introduction to All of Us Research Program

**Topic 10**

- Closing or following up to schedule appointment

**Topic 12**

- COVID-19 protocols for in-person visit

**Topic 22**

- Automated voicemail prompt

**Topic 24**

- Scripted voicemail message

**Topic 27**

- Update or retention calls

**Topic 29**

- Explaining precision medicine or need for representation

**Topic 33**

- Describing physical measurements visit procedure

**Topic 41**

- Working through objections or barriers to participation

### Topic Prevalence as a Function of Enrollment or Participation

#### Overview

**Figure 3:**
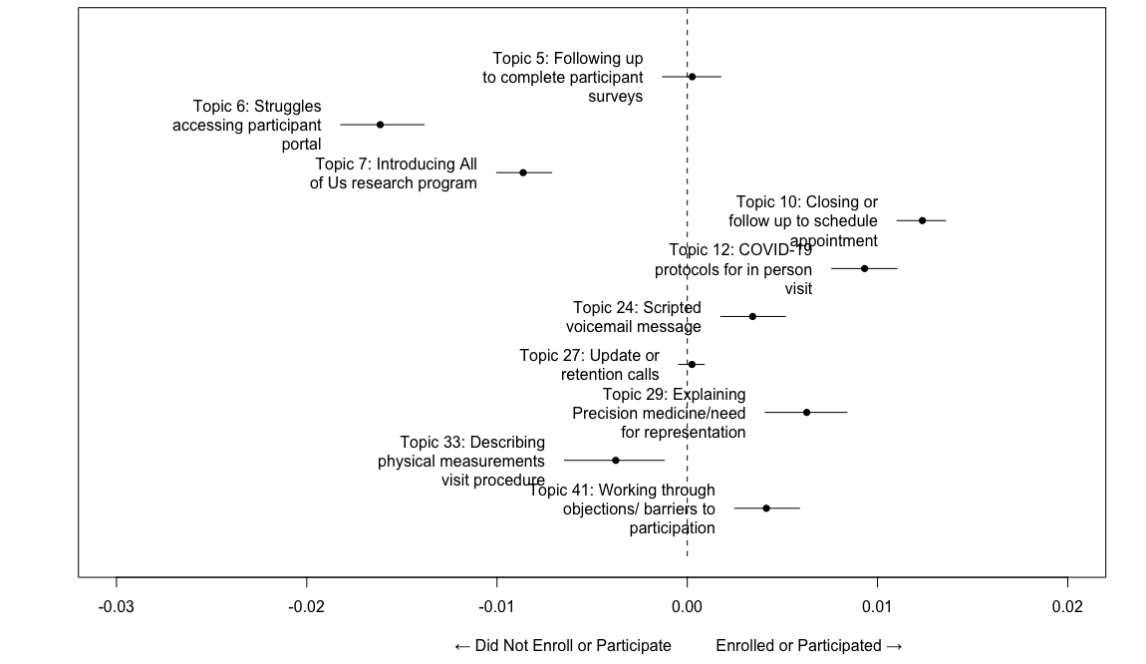
Topic proportions as a function of enrollment into the All of Us Research Program.

Figure 3 plots the change in topic proportion when comparing conversations where the research participant enrolled in the research study to those who did not. That is, what is the likelihood of a topic being brought up in a conversation between RAs and participants as a function of the recruitment outcome? The plots also include confidence intervals indicating whether mean differences in prevalence are statistically significant.

#### Enrolled or Participated Group

Our results, shown in Textbox 2, suggest that recruitment calls in which RAs follow-up to close participants and schedule appointments (Topic 10), as well as describing COVID-19 protocols for in-person visits (Topic 12), are more likely to appear in the enrolled group. Illustrative talk turns include, “I’m calling regarding your enrollment into the All of Us research program,” “So we started the process and I was wondering if you’d like to go ahead and schedule an appointment to come in for the other part of the enrollment process,” “once you come in security will alert that you are here, we come to you do a rapid test that takes 10 to 15 minutes. Once its negative they will bring you into the building,” and “everybody has to take a COVID test before you come into samples.” These results are as expected, given that these topics of conversation are more likely to occur in interactions in which participants have indicated some level of interest in participation.

We also found that scripted voicemails left by RAs (Topic 24) are also more likely to be associated with successful enrollment. The template used was, “Hi this is [research assistant’s name] and I’m calling from Morehouse School of Medicine and today we’re calling to let people know about the All of Us research study. It’s a nationwide study that has a goal of recruiting one million participants to push forward the initiative of precision medicine, that is the type of medicine that uses information about a person’s lifestyle, their environment, and biology to figure out new ways to treat, cure and prevent different types of diseases. If this sounds like something that you are interested in, please feel free to give us a callback.” This result suggests that RAs who left voicemails for participants who did not answer initial calls were more likely to get callbacks and ultimately, successful enrollment.

Another topic that was more likely to occur in recruitment calls that concluded in successful enrollment was the explanation of the purpose of the study, precision medicine, and the need for representation (Topic 29) to prevent and address health disparities. In one high-scoring utterance on this topic, the RA states in response to what the purpose of the study is: “So precision medicine is trying to develop health care treatments on a more individual basis instead of the sort one size fits all mentality,” in another utterance the RA states: “Our goal as a program is to get more participants from across the country with different background, lifestyle, and genetics because you so know so many things can affect health.” Upon further review, it appears characteristic utterances on this topic follow a template that begins with a layman’s definition of precision medicine followed by a description of the goal of the AoURP. The increased likelihood of detecting this topic in successful recruitment calls may indicate that describing the purpose of the study is an important factor in potential participants’ enrollment decision-making process.

Finally, and most insightful, the model identified a topic that reflected objections and barriers to participation (Topic 41). This topic had the highest expected proportion in the entire corpus, indicating that it was the most common topic or theme across the entire corpus (after Topics 2 and 22, ie, automated voicemail prompts). In one exchange, a participant inquired about participation costs, “How much is all of these?” In response, the RA provided reassurance that “You don’t have to pay anything, we don’t look at your insurance or have to change doctors.” In another exchange, a participant tells the RA they are “skeptical” and “scared” because they “already got the COVID-19 shots and I’ve been having some bad headaches constantly after that.” The RA responds, “Well I assure you that we have nothing in our needle, we are just taking a small blood sample and we’re not putting anything in your bloodstream.” We found other examples of participants being cautious about “not knowing” what will be done with their samples once collected and assurances from RAs that they would not be given any treatments. Other challenges brought up included transportation, for example, “I can’t participate because I don’t have transportation,” suspicion toward RAs, for example, “How did you get my name and number?” and mistrust based on past negative experiences with discrimination related to health, for example, “I was one of the people that was exposed. They refused to tell me what types of toxic chemicals or materials they were working with.” In one final illustrative exchange, a potential participant asks about the results of his blood sample, “You took some blood and you analyze that, can I have the results?” The RA responds that the “Sample [is] shipped to a secure facility by mail and that the facility would be doing” genetic analysis.

**Illustrative talk turns for topics associated with increased likelihood of enrollment.**

**Topic 10: Closing or following up to schedule appointment**

Research assistant (RA): “I’m calling regarding your enrollment into the All of Us research program,” “so we started the process and I was wondering if you’d like to go ahead and schedule an appointment to come in for the other part of the enrollment process.”

**Topic 12: COVID protocols for in-person visit**

RA: “Once you come in security will alert that you are here, we come to you do a rapid test that takes ten to fifteen minutes. Once its negative they will bring you into the building,” and “everybody has to take a COVID test before you come into samples.”

**Topic 24: Scripted voicemail message**

RA: “Hi this is [research assistant’s name] and I’m calling from Morehouse School of Medicine and today we’re calling to let people know about the All of Us research study. It’s a nationwide study that has a goal of recruiting one million participants to push forward the initiative of precision medicine, that is the type of medicine that uses information about a person’s lifestyle, their environment, and biology to figure out new ways to treat, cure, and prevent different types of diseases. If this sounds like something that you are interested in, please feel free to give us a callback.”

**Topic 29: Explaining precision medicine or need for representation**

RA: “So precision medicine is trying to develop health care treatments on a more individual basis instead of the sort one size fits all mentality”

RA: “Our goal as a program is to get more participants from across the country with different background, lifestyle, and genetics because you so know so many things can affect health.”

**Topic 41: Working through objections or barriers to participation**

Participant: “How much is all of these?” RA: “You don’t have to pay anything, we don’t look at your insurance or have to change doctors.”

Participant: “Already got the COVID shots and I’ve been having some bad headaches constantly after that.” RA: “Well I assure you that we have nothing in our needle, we are just taking a small blood sample and we’re not putting anything in your bloodstream.”

#### Did Not Enroll Group

Illustrative talk turns of high-ranking topics among those who did not enroll are displayed in Table 2. The first topic that is more likely to be prevalent in the did not enroll group is instructions to accessing or challenges accessing the participant portal (Topic 6). Illustrative examples of talk turns in Topic 6 include instances of RAs “sending emails [with] instructions on how to register” for the research study, as well as “email[s] with the link to the [All of Us] website for more information and also a link to start an account if [the participant is] ready for that.” We also found many examples of RAs walking participants through challenges accessing their portals once created. In one exchange, an RA helps the participant search for the correct username when trying to access the portal via their phone: “Let’s try your phone number as your username, then we click on forgot password, a link should be sent to your phone.” Utterances like “if you do not remember your password, click forgotten password. Let’s see what happens,” were very common in this topic.

Our results also suggested that scripted introductions to the AoURP (Topic 7) were also more likely to occur in the not enrolled group. There is an overlap between this topic and Topic 24. Both include high-scoring scripted utterances like, “Hi yes like I said this is [Name]. I’m calling from Morehouse School of Medicine today we’re reaching out the members of the community to talk about the “all of us research program” and describe the purpose of the “research study called the all research program to speed up medical breakthroughs.”

Finally, we found that the descriptions of the physical measurements visit (Topic 33) were also more likely to occur in the did not enroll group. In one exchange, an RA explains that by participating, “You would complete three maybe four surveys ... then we would bring you in for the second part which we would take measurements of your height, weight, blood pressure, and you would provide a small blood sample.” The participant responded that they feel comfortable about completing surveys about “My demographic, but I don’t know if I wanna come in and give a blood sample and things of that nature. I also would like to say that I do understand the importance of these things [but] I’m not too comfortable with that.” In other exchanges, “atrocities that have happened” were alluded to as were “caregiver” responsibilities that would make the participant unable to “contribute” to the “second half ... where we would take your measurements, your height, your weight, your blood pressure, and you would provide small urine and blood samples,” even in light of “cash compensation for participation.” Conversations on this topic suggest that participants who did not enroll were more likely to feel a burden of participation related to the second part of the study.

**Table 2:** Illustrative talk turns for topics associated with a decreased likelihood of enrollment.

Topic	Illustrative talk turns
Topic 6: Instructions or struggles accessing participant portal	RA^a^: “sending emails [with] instructions on how to register” for the research study as well as “email[s] with the link to the [All of Us] website for more information and also a link to start an account if [the participant is] ready for that.” RA: “Let’s try your phone number as your username, then we click on forgot password, a link should be sent to your phone.”
Topic 7: Scripted introduction to All of Us Research Program Topic 24: Scripted voicemail message	RA: “Hi yes like I said this is [Name]. I’m calling from Morehouse School of Medicine today we’re reaching out the members of the community to talk about the “all of us research program” and describe the purpose of the “research study called the all research program to speed up medical breakthroughs.”
Topic 33: Describing physical measurements visit procedure	RA: “You would complete three maybe four surveys ... then we would bring you in for the second part which we would take measurements of your height, weight, blood pressure, and you would provide a small blood sample.” Participant: “My demographic, but I don’t know if I wanna come in and give a blood sample and things of that nature. I also would like to say that I do understand the importance of these things [but] I’m not too comfortable with that.”

^a^RA: research assistant.

#### Both Groups

Two topics were equally likely to show up in both the enrolled and not enrolled groups. The first topic is RAs following up with participants to complete surveys (Topic 5). These surveys included initial “demographic” surveys, as well as the “second part of the study whereby we schedule you to come in and we do your physical measurements, blood, urine sample so ... I was wondering if you’re ready for that part however you would need to complete your survey online.” In these exchanges, RAs were usually following up with participants about “completing [their] surveys” before “coming in for the appointment.” Finally, we identified a topic around follow-up calls, including updates and retention calls. We identified three scenarios where RAs were calling. In the first situation, RAs were following up with participants who indicated an interest in enrolling but did not complete consent forms indicating some level of interest in participation but not following through on any level of participation (eg, demographic surveys, physical measurements, and visits). In the second situation, research participants consented but did not complete surveys, the first level of participation. These two first scenarios reflect RAs following up with participants requesting updates to encourage further engagement with the AoURP. In the final situation, participants completed surveys and physical measurement visits and were followed up with to complete a retention survey that was introduced as a required component in the later part of recruitment by the National Institutes of Health for All of Us enrollment centers.

## Discussion

### Principal Findings

Since its inception, MSM has developed and refined a multilevel approach to engagement with the community. This results in significant success in recruiting and enrolling members of the African American community in research. As a result of this success, MSM was selected to participate in the AoURP as part of a larger consortium, the SEEC in 2018. The COVID-19 pandemic accelerated the digital transformation of our clinical trial recruitment platform, which created an unprecedented opportunity to study recruitment using real-world data on the content of conversations between RAs and potential participants. The goal of this analysis is to report a preliminary investigation of these conversations.

In analyzing the metadata of these calls, we found that the average number of calls per participant (2.90 vs 1.48), as well as the average length of conversations (64.28 vs 25.23) for the enrolled group, were higher than that of the not enrolled group. These findings align with research in sales and marketing increased frequency and depth of contact with potential participants correlate with increased likelihood of “closing” [22,23]. Using automated content analysis methods, we identified topics that reflected challenges associated with participation in clinical trials by underrepresented community members.

These findings are in line with existing literature that highlights time and resource constraints, including financial burden, time commitment, transportation, and compensation related to trust as barriers to clinical trial participation [24,25]. Notably, our analysis did not highlight unique barriers or objections specific to African American communities, instead reflecting common themes identified that were consistent across many UBR communities. Beyond barriers, we also identified topics that reflect concerns around clinical trial participation, including mistrust of investigators and the clinical trial process, as well as a lack of awareness about the purpose and value of trials [25,26]. African American mistrust of the US health care system and medical research is based both on historical research misconduct, as well as ongoing systemic issues and misinformation [27]. Interestingly, we also found that where concerns were addressed and RAs worked with potential participants to work through objections, participation was much more likely.

Finally, we found that “scripted language” is common between the enrolled and not enrolled groups but the differences are within the content of those scripts. The main difference seems to be that Topic 7 (scripted introduction to AoURP) is an introduction when someone picks up the phone while Topic 24 (scripted voicemail message) is someone leaving a message. There may be some selection bias here because those who listened to the voicemail and were interested in the study called back while those who did not find the study interesting did not. It is also likely that most folks who reached out did not find the study interesting, and therefore, did not enroll, which would push this topic to the did not enroll category.

This study is consistent with previous studies that consider topical framing and placement as key predictors of successful clinical trial recruitment. A few studies have focused on information exchange between members of the clinical research team and potential study volunteers is a critical influence on research volunteers’ journey from awareness to enrollment. This includes Eggly et al [28], who used linguistic analysis to explore offers to participate in clinical trials. The authors identified differences in the quality of the interactions and quantity of the interactions between physicians and African American patients versus White patients. In another study of strategies to persuade participation in cancer clinical trials, Barton et al [12] conducted a discourse analysis of 22 oncology trial-related interactions stratified by race and argued that topical framing and placement influence momentum toward participation. However, these prior studies are limited by sample size and the extent they examined informational dimensions of offers to participate in clinical trials—such as descriptions of the procedures of the trial and discussions of the trial regimen in comparison with standard treatment.

The results of our STM approach to analyzing recruitment calls suggest that natural language processing is a promising method that reliably identifies latent topics in conversations with minimal human subjectivity. This not only permits the establishment and analysis of larger data corpora but also enables hypothesis testing through the incorporation of metadata into models. This enables relationship estimations between metadata and topical content and prevalence.

### Limitations

The limitations of our analysis are 3-fold. First, we had limited information on the demographics of potential participants who did not enroll or participate. This limited our ability to include demographics including race and gender of these potential participants in our topic model estimation. Though this missingness likely did not influence the topics that were identified, more complete demographic data would have allowed us to produce unbiased estimates of associations between demographics and topical prevalence, that is, the likelihood of certain topics being brought up. Second, while a noninsignificant proportion of recruitment conversations are scripted, the tone and tenor of the recruiter matters for how information is conveyed and received. That is, not only what clinical RAs and coordinators say, but how they say it influences how information is received by potential participants. Our analysis only captures what is being said, that is, topics. Future research should consider methods for capturing sentiment. Additionally, given gender differences in communication, analyses of the gender of RAs on the content and trajectory of recruitment calls and subsequent enrollment rates could provide valuable insights into future studies. Finally, we found many errors during the transcription of audio to text by ObserveAI, which may indicate bias in proprietary natural language processing algorithms when being applied to audio generated from African American speakers [29]. More culturally congruent speech-to-text algorithms are needed.

### Conclusions

The goal of this analysis is to analyze conversations between RAs and community members who were recruited to participate in the AoURP. This type of work supports the building of an evidence-based framework to guide recruitment conversations. The use of this method of analysis addresses the need for critical data to support the development of the science of inclusion, clinical research diversity, and community engagement.

## Abbreviations

AoURP: All of Us Research Program
EHR: electronic health record
gRoR: genomic return of results
MSM: Morehouse School of Medicine
PPI: participant-provided information
RA: research assistant
SEEC: Southeast Enrollment Center
STM: structural topic modeling
UBR: underrepresented in biomedical research

## Data Availability

The datasets generated or analyzed during this study are not publicly available due to the sensitive nature of call transcripts but are available from the corresponding author upon reasonable request.
